# Tracking the Emergence of Conceptual Knowledge during Human Decision Making

**DOI:** 10.1016/j.neuron.2009.07.030

**Published:** 2009-09-24

**Authors:** Dharshan Kumaran, Jennifer J. Summerfield, Demis Hassabis, Eleanor A. Maguire

**Affiliations:** 1 Wellcome Trust Centre for Neuroimaging, Institute of Neurology, University College London, 12 Queen Square, London WC1N 3BG, UK; 2 Institute of Cognitive Neuroscience, University College London, 11 Queen Square, London WC1N 3BG, UK; 3 Department of Psychology, Stanford University, Stanford, CA 94305, USA

**Keywords:** SYSNEURO

## Abstract

Concepts lie at the very heart of intelligence, providing organizing principles with which to comprehend the world. Surprisingly little, however, is understood about how we acquire and deploy concepts. Here, we show that a functionally coupled circuit involving the hippocampus and ventromedial prefrontal cortex (vMPFC) underpins the emergence of conceptual knowledge and its effect on choice behavior. Critically, the hippocampus alone supported the efficient transfer of knowledge to a perceptually novel setting. These findings provide compelling evidence that the hippocampus supports conceptual learning through the networking of discrete memories and reveal the nature of its interaction with downstream valuation modules such as the vMPFC. Our study offers neurobiological insights into the remarkable capacity of humans to discover the conceptual structure of related experiences and use this knowledge to solve exacting decision problems.

## Introduction

The capacity to bring prior knowledge to bear in novel situations is a defining characteristic of human intelligence. A powerful way in which humans achieve this is through the use of concepts, which are formed through abstraction, and capture the shared meaning of similar entities through an organizing principle that explains their relatedness. For instance, although an Alsatian and a Chihuahua look perceptually very different, we can easily appreciate that they have similar attributes (e.g., they bark) because they can be recognized as instances of a particular concept, in this case a dog (Locke, 1690; Martin, 2007; Murphy, 2004; Rogers and McClelland, 2004). While the devastating consequences of the degradation of well-established conceptual representations are all too apparent in neurological conditions like semantic dementia (Patterson et al., 2007), little is known about the neural mechanisms underpinning the emergence of conceptual knowledge, its application in novel settings, and its influence on human decision making (Shea et al., 2008).

Empirical research to date in the fields of memory and decision making has tended to focus on discovering the neural mechanisms mediating memory for our unique experiences from our past (i.e., episodic memory; Davachi, 2006; Eichenbaum, 2004; Eichenbaum et al., 2007) and for the reward value of individual stimuli and actions, the latter captured successfully by reinforcement learning (RL) algorithms (e.g., Montague et al., 2006; Rangel et al., 2008). While a collection of isolated memories or reward associations may allow simple inferences to be made through the operation of logical reasoning processes at retrieval (e.g., transitive inferences; Greene, 2007; Preston et al., 2004), the capacity for efficient generalization in novel situations is limited. What is needed, therefore, is a neural system that abstracts the commonalities across multiple related experiences, thereby creating a network of conceptual knowledge that captures the higher-order structure of the environment. While the hippocampus has often been cast as a key player in the emergence of conceptual knowledge (Cohen and Eichenbaum, 1993; Eichenbaum, 2004) and the broader notion of semantic memory, empirical data have provided only equivocal support for this idea (e.g., Duff et al., 2006; Eichenbaum, 2004; Vargha-Khadem et al., 1997). As such, whether the hippocampus, or instead neocortical areas within the medial temporal lobe (MTL) or prefrontal cortex (PFC) (Cohen and Eichenbaum, 1993; Eichenbaum, 2004; McClelland et al., 1995; Miller et al., 2002; Norman and O'Reilly, 2003; Vargha-Khadem et al., 1997), are critical to conceptual learning remains an unresolved question in neuroscience.

**Figure 1:**
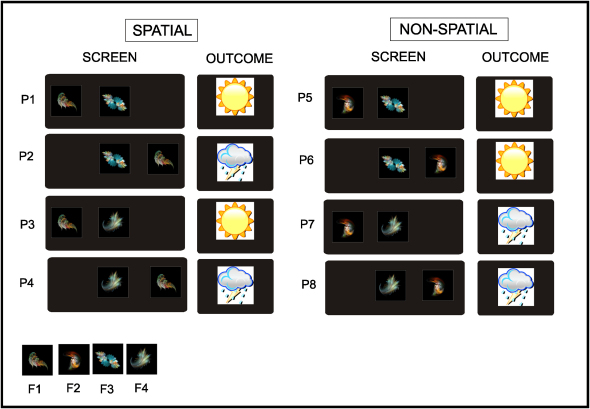
Experimental Design Subjects were instructed to play the role of a weather forecaster and try to learn over the course of the experiment how each one of eight “patterns” of shapes on the screen (P1–P8) were deterministically associated with one of two outcomes: sun or rain (see Experimental Procedures). Since all eight patterns were made up of the same four fractals (F1–F4), subjects were required to use associative information (i.e., shape-location, shape-shape conjunctions) to perform successfully. Importantly, while participants could simply learn the correct response associated with a given pattern in isolation (e.g., P1 = sun; P2 = rain), they could also acquire spatial and nonspatial conceptual knowledge. In this way, participants could recognize that individual patterns (e.g., P1, P3) constitute instances of a particular concept (i.e., F1 = left), allowing them to disregard unimportant differences between them (i.e., the identity of the central fractal) and appreciate their shared meaning (i.e., outcome: sun). Subjects were not explicitly told about the spatial and nonspatial structure of the task and had to acquire this through learning. There were two experimental sessions, Initial and New, which shared a similar underlying conceptual structure but differed in terms of the set of fractals used. Fractals were used as stimuli, rather than real-life objects, to investigate the learning of new concepts without contamination from previous exposure (see Experimental Procedures).

**Figure 2:**
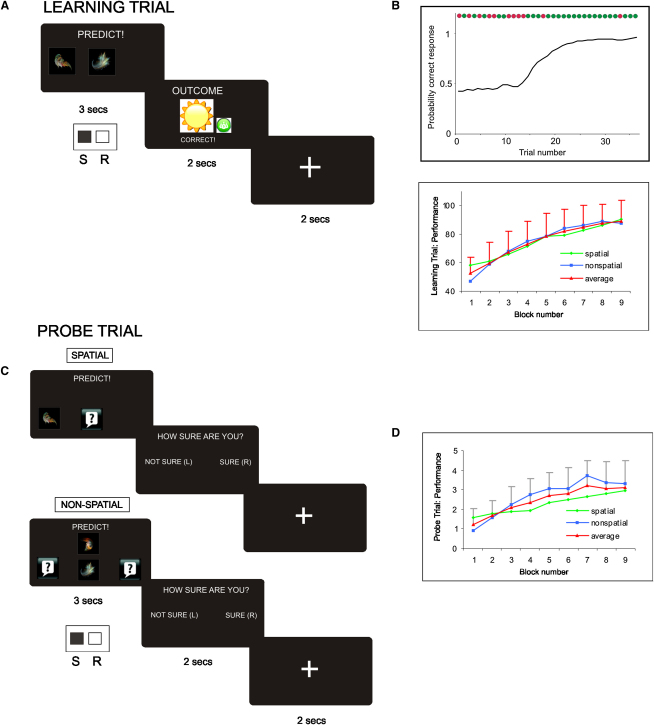
Learning and Probe Trials: Timeline and Behavioral Data (A) Learning trial. Participants viewed a pattern on the screen (3 s), entered their prediction (using index/middle finger), and received feedback concerning actual outcome (sun or rain: 2 s), their correctness (correct/incorrect), and reward (i.e., whether they had won or lost money). Presentation of all eight patterns (P1–P8) was pseudorandomly intermixed during learning trials. (B) Learning trial behavioral data. Upper panel: an example of an individual participant's learning curve estimated by the state-space model (Smith et al., 2004), which shows the probability of a correct response for an individual pattern as a function of trial number. Binary performance is shown above (green circle = correct, red circle = wrong). Lower panel: block-by-block performance (percent correct responses) of the entire group (n = 25) during the Initial session. Performance on spatial patterns (P1–P4) is shown in green, nonspatial patterns (P5–P8) in blue, and averaged performance in red. Error bars denote standard deviation. (C) Probe trial. Participants were required to make a prediction (index/middle finger) based on partial patterns (3 s). No feedback was provided, but participants were rewarded for correct predictions at the end of the experiment. Participants also provided confidence ratings (2 s) by indicating whether they were “sure” or “not-sure” by button press. In spatial probe trials (upper panel), one fractal (i.e., either F1 [illustrated] or F2) was presented on either the left (illustrated) or the right. A question mark displayed in the central position indicated that the identity of the central fractal was not known. In nonspatial probe trials (lower panel), one fractal was presented in the central position (F3 or F4 [illustrated]), with another above (F1 or F2 [illustrated]). The question mark indicated that the position of the peripheral fractal was not known. Two varieties of probe trials were included: “outcome determined” and “outcome undetermined” (Figure 5). In outcome-determined trials, the main trials of interest, participants could deploy conceptual knowledge to make accurate predictions: e.g., the presence of F1 on the left in a spatial probe trial is predictive of sun, regardless of the identity of the central shape. (D) Probe trial. Block-by-block probe performance of entire group (n = 25) during Initial session. Performance on spatial probe trials shown in green, nonspatial in blue, and averaged performance in red. Error bars denote standard deviation.

To address these questions, we constructed a paradigm based on a task known to be dependent on the hippocampus from a previous neuropsychological study (Kumaran et al., 2007). In our task, participants were instructed to play the role of a weather forecaster and learn over multiple trials how each of eight patterns was (deterministically) associated with one of two outcomes (i.e., sun or rain) (Figure 1). During “learning” trials, participants viewed a pattern on the screen, entered their prediction (using index/middle finger), and received feedback concerning actual outcome (sun or rain), their correctness (correct/incorrect), and reward (i.e., whether they had won or lost money) (Figure 2A). Since all eight patterns were constructed from the same four fractals, successful performance required participants to use associative information consisting of shape-location and shape-shape conjunctions rather than elemental information (e.g., single shape) as studied previously (Montague et al., 2006; Toni et al., 2001).

Critically, while participants could simply learn the correct response associated with a given pattern in isolation (e.g., pattern 1 = sun, pattern 2 = rain), there was also the opportunity for them to acquire spatial and nonspatial conceptual knowledge, which was assessed in probe trials at the end of each learning block and using a debriefing protocol (Figure 2C). Specifically, participants could learn that fractal 1 predicts sun when on the left, and rain when on the right, irrespective of the identity of the central shape (i.e., fractal 3 or 4), by abstracting the commonalities across the relevant patterns, therefore termed “spatial” (i.e., P1–P4) (Figure 1). In a similar vein, participants could learn that the shape-shape combination of fractals 2 and 3 predicts sun, and 2 and 4 rain, regardless of the position of fractal 2, by appreciating the relationship between the relevant “nonspatial” patterns (i.e., P5–P8). In this way, participants could recognize that individual patterns (e.g., P1, P3) constitute instances of a particular concept (i.e., F1_left_), allowing them to disregard unimportant differences between them (i.e., the identity of the central fractal) and appreciate their shared meaning (i.e., outcome: sun). By understanding the task structure in this fashion, participants could generalize successfully when confronted with partial patterns during probe trials (e.g., F1_left_: Figure 2C), which provided us with an online measure of the level of conceptual knowledge acquired throughout the experiment. Furthermore, participants could use knowledge of this sort as an effective guiding framework (i.e., schema) for accelerating learning in a New session, where the stimuli were perceptually novel but the underlying conceptual structure similar.

Our experimental design, therefore, incorporated three key aspects: the learning of individual associations (i.e., between patterns and outcomes), the emergence of conceptual knowledge through the abstraction of common features between patterns related through shared meaning, and a transfer test where participants' ability to use this knowledge as a schema (Bartlett, 1932; Tse et al., 2007) in a perceptually novel setting (i.e., the New session) was assessed. In contrast, previous studies have investigated how participants classify stimuli based on their physical resemblance (Ashby and Maddox, 2005), elemental value (e.g., the “standard” weather prediction task [Knowlton and Squire, 1994; Poldrack et al., 2001]), or simple unidimensional rules (e.g., color) (Ashby and Maddox, 2005) rather than a higher-order conceptual structure. Of note, learning in such perceptual categorization tasks, and also artificial grammar paradigms, proceeds largely independently of the hippocampus and wider MTL, often being predominantly implicit in nature and involving regions such as the striatum and lateral PFC (Ashby and Maddox, 2005; Knowlton and Squire, 1993; Knowlton et al., 1994; Poldrack et al., 2001; Reber, 1967; Strange et al., 1999).

## Results and Discussion

### Behavioral Data

At a behavioral level, the improvement in participants' performance on learning trials during the Initial session (Figure 2B) was paralleled by the emergence of spatial and nonspatial conceptual knowledge indexed by probe trials (Figure 2D). Importantly, conceptual knowledge exerted a significant influence on participants' choices during learning trials, with probe trial performance correlating significantly with participants' choices on a given learning trial (p < 0.01: see below). Further, performance on learning trials involving an individual pattern (e.g., P1) showed a greater correlation with performance on other patterns within a domain (i.e., spatial: P2–P4), as compared to across domains (i.e., nonspatial: P5–P8) (t = 2.2, p = 0.05), as would be expected if participants integrated information across relevant patterns (see Experimental Procedures).

The assertion that probe trial performance is guided primarily by conceptual knowledge (e.g., F1_left_ means sun regardless of the identity of the central fractal) rather than the retrieval of multiple individual associative pairings (i.e., P1 = sun, P3 = sun) receives support from several features of the behavioral data. First, probe trial performance showed a robust correlation with a composite score obtained from a debriefing protocol (r = 0.65, p < 0.001: see Supplemental Experimental Procedures), which assessed participants' ability to express and deploy conceptual knowledge in a context quite different from the original learning situation. Importantly, in a separate follow-up behavioral experiment, where participants provided verbal descriptions of the conceptual structure of the task after each learning block (see Supplemental Results and Supplemental Experimental Procedures), the correlation between probe performance and task structure descriptions was highly significant (r = 0.71, p < 0.001) and remained significant (p < 0.05) after the effect of learning trial performance had been partialled out (r = 0.55, p < 0.001). Second, the magnitude of difference in reaction times between probe trials and learning trials in the fMRI experiment was small, though significant (1.52 versus 1.38 s; t = 3.8, p = 0.001), arguing against the notion that probe performance is supported by the retrieval of multiple individual associative pairings (e.g., see Shohamy and Wagner, 2008). Finally, probe trial performance during the Initial session also showed a significant correlation with participants' performance in the new session, after initial session performance had been covaried out (r = 0.41, p = 0.03: see below), in line with the assertion that probe trials index conceptual knowledge which mediates transfer to a perceptually novel setting.

### Neuroimaging Data

#### Initial Session: Brain Areas Associated with Proficient Performance during Learning Trials

Given behavioral evidence that participants had acquired conceptual knowledge, we next turned to the fMRI data acquired during the Initial session. Since we did not observe any significant differences as a function of domain (i.e., spatial versus nonspatial), even at liberal thresholds (i.e., p < 0.01 uncorrected), we collapsed across this factor for all subsequent analyses (see Supplemental Results). We first conducted an analysis designed to identify the overall brain network associated with proficient performance on learning trials. As a first step, we set out to convert participants' binary performance data, where 1 indexed a correct response and 0 an incorrect response, into trial-by-trial estimates of the probability of a correct response for each pattern (i.e., a learning curve). To achieve this, we employed a dynamic estimation technique that has previously been used to correlate neural activity with binary performance data during learning experiments in monkeys (Wirth et al., 2003) and during human fMRI (Law et al., 2005), termed the state-space model (Smith et al., 2004) (see Supplemental Experimental Procedures). A significant advantage of this technique, in comparison to related approaches (e.g., reinforcement learning [RL] models), is that it allows variations in the shape of individual learning curves to be effectively captured. For instance, it is well recognized that individual learning curves often show an abrupt transition from low to asymptotic levels of performance (e.g., Figure 2B, upper panel) (Gallistel et al., 2004), even though group-averaged curves show gradually increasing performance (e.g., Figure 2B, lower panel).

To verify that the state-space model provided a better fit to the binary choice data observed in our experiment, we performed comparisons with a standard RL model (Q-learning; Watkins and Dayan, 1992) and the moving average method (see Supplemental Experimental Procedures; Smith et al., 2004) using a standard approach (i.e., by calculating mean squared errors for each model). For our data set, the state-space model generated closer fits to the observed data compared to these two other approaches (see Supplemental Experimental Procedures), which accords with previous validations in the context of associative learning tasks (Smith et al., 2004).

**Figure 3:**
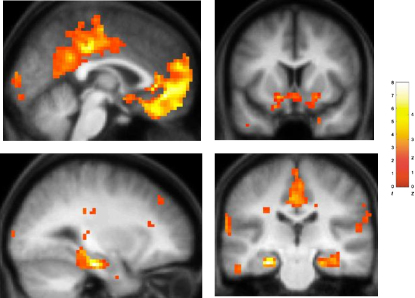
Brain Areas Associated with Proficient Performance during Learning Trials in the Initial Session Brain areas whose activity during learning trials shows a significant positive correlation with a participant-specific index of performance (probability_success). The probability_success parametric regressor was created by converting participants' binary choice data into estimated learning curves (Figure 2B) using the state-space model (see Supplemental Experimental Procedures). Activations are shown on the averaged structural MRI scan of the 25 participants, with the color bar indicating the t statistic associated with each voxel and the z score equivalent. Activations in vMPFC and PCC are shown in sagittal section (upper left panel). Upper right panel: coronal section showing activation in bilateral ventral striatum. Lower left panel: sagittal section showing activation in the left parahippocampal cortex extending into hippocampus. Lower right panel: coronal section showing activation in PCC, bilateral parahippocampal cortex extending into hippocampus. See Table S1 for a full list of activations. Activations in left parahippocampal cortex, amygdala, vMPFC, and PCC were significant at p < 0.05 FWE corrected (see Experimental Procedures). Activations are shown at p < 0.005 (uncorrected) for display purposes.

The state-space model, therefore, allowed us to create participant-specific trial-by-trial parametric regressors (“probability_success”: see Experimental Procedures) that we used to regress against the learning trial fMRI data. Our results show that activity in brain regions including parahippocampal cortex, amygdala, posterior cingulate cortex (PCC), ventral striatum, and ventromedial prefrontal cortex (vMPFC) was significantly correlated with the probability of success (Table S1 and Figure 3). Given that participants were rewarded for a correct prediction on each trial in our paradigm, both with positive feedback and money, these findings are consistent with previous work suggesting that these brain regions form part of a neural system coding value predictions that guide choice behavior (Montague et al., 2006). We also conducted a number of supplemental analyses, which effectively excluded alternative explanations for the activation of this brain network (see Supplemental Results). Our results, therefore, provide insights into how brain regions traditionally associated with memory in the medial temporal lobe (MTL), such as the parahippocampal cortex, become engaged in a decision-making context when value predictions must be based on associative (i.e., shape-shape, shape-location) rather than more simple elemental information as studied previously (Montague et al., 2006).

#### Initial Session: Functionally Coupled Activity in Hippocampus and vMPFC during Learning Trials Tracks the Emergence of Conceptual Knowledge

While this analysis reveals the overall network engaged when participants perform proficiently on learning trials, it does not dissociate between brain regions involved in memory for individual associative pairings and those supporting conceptual representations. To identify the neural circuitry specifically underpinning the emergence of conceptual knowledge and its influence on choice behavior, we used participants' performance on probe trials as leverage with which to interrogate the learning trial fMRI data (see Experimental Procedures; Figure 2D). We therefore created a vector, termed “probe_performance,” which was entered as a second parametric regressor against the relevant learning trials during the preceding learning block (see Experimental Procedures). Importantly, this trial-by-trial probe_performance vector was a robust indicator at a behavioral level of whether a participant's response on a given trial was correct or incorrect (r = 0.42, p < 0.001), even once the (highly significant: r = 0.49, p < 0.001) effect of the probability_success vector had been covaried out (p < 0.01).

**Figure 4:**
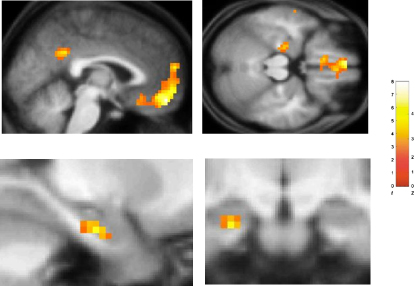
Brain Areas Tracking the Emergence of Conceptual Knowledge during Learning Trials in the Initial Session Brain areas whose activity during learning trials showed a significant positive correlation with a participant-specific index of conceptual knowledge acquisition (probe_performance), after the effect of probability_success had been covaried out. Activations are shown on the averaged structural MRI scan of the 25 participants, with the color bar indicating the t statistic associated with each voxel and the z score equivalent. Activation in the left hippocampus is shown in axial section (upper right panel) and in close-up in lower left (sagittal) and lower right (coronal) panels. Activation in vMPFC and PCC are shown in sagittal section (upper left panel). See Table S2 for a full list of activations. Activation in hippocampus significant at p < 0.001 uncorrected and p < 0.05 SVC corrected (see Experimental Procedures). Activation in vMPFC significant at p < 0.05 FWE corrected. Activations are shown at p < 0.005 for display purposes.

We next sought to identify brain regions where neural activity on a given trial selectively tracked the emergence of conceptual knowledge, above and beyond any correlation with probability of success. To effect this analysis, we entered both probability_success and probe_performance vectors as parametric regressors modulating neural activity during learning trials within the same general linear model (see Experimental Procedures). Strikingly, activation within the left hippocampus, vMPFC, and PCC showed a robust positive correlation with probe performance, even after any effect of probability of success had been covaried out (Figure 4 and Table S2). In marked contrast, no significant activation was observed in other areas previously identified to show a correlation with the probability of success, including the parahippocampal cortex, even at liberal thresholds (i.e., p < 0.01 uncorrected). Indeed, activity in bilateral parahippocampal cortex (anatomical region-of-interest [ROI] analysis: see Experimental Procedures) showed a significantly greater correlation with the probability of success, as compared to probe performance (p < 0.05). As such, our findings are consistent with the notion that areas such as the parahippocampal cortex play a greater role in memory for individual associative pairings, perhaps involving the formation of configural or unitized representations, as has previously been hypothesized (Cohen and Eichenbaum, 1993; Haskins et al., 2008; Mayes et al., 2007; Rudy and Sutherland, 1989). In this way, the parahippocampal cortex may support the capacity of patients with amnesia and hippocampal damage to perform relatively well (i.e., 75% correct responses), though not normally, on a similar task, without developing conceptual knowledge of the task structure (Kumaran et al., 2007).

Thus far, our findings provide behavioral evidence that conceptual knowledge is acquired gradually during learning, plays a significant role in guiding participants' choices, and is underpinned by neural activity in hippocampus, posterior cingulate cortex (PCC), and vMPFC. We next set out to test the hypothesis that the hippocampus and vMPFC, two reciprocally interconnected brain regions (Ongur et al., 2003), interact during the emergence of conceptual knowledge during decision making. To achieve this, we used a psychophysiological interaction (PPI) analysis, which assesses whether the functional coupling of distant brain regions varies according to experimental parameters (Friston et al., 1997) (see Experimental Procedures). This enabled us to ask whether the left hippocampus, our source region, significantly influenced activity in vMPFC, specifically in relation to the level of conceptual knowledge acquired (i.e., condition × probe_performance interaction). We observed a significant correlation between neural activity in left hippocampus and the functionally defined vMPFC region (t = 1.9, p = 0.03), but not PCC (p > 0.1), whereby greater conceptual knowledge was associated with stronger coupling. This finding provides evidence that the hippocampus and vMPFC act as a circuit during the acquisition and application of conceptual knowledge during decision making.

#### Initial Session: Neural Activity in Hippocampus and vMPFC Correlates with Performance during Probe Trials

**Figure 5:**
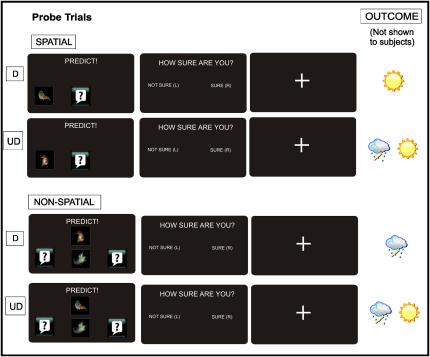
Design of Probe Trials We included two varieties of probe trials termed “outcome_determined” (labeled “D” and “outcome_undetermined” (labeled “UD”). In outcome-determined trials, the main trials of interest, participants could deploy conceptual knowledge to make accurate predictions: specifically, in spatial probe trials of this type, the presence of F1 on the left is predictive of sun, regardless of the identity of the central shape. In nonspatial outcome_determined probe trials, the presence of fractals F2 and F4 on the screen (shown) is indicative of rain, regardless of the position of F2. In outcome_undetermined probe trials, however, conceptual knowledge could not be deployed, and the outcome could not be predicted based on the information given (i.e., 50% sun, 50% rain). These trials, however, were otherwise closely matched to outcome-determined trials, providing an appropriate comparison condition for the neuroimaging analyses, as well as serving the function of preventing participants from gaining information about the higher-order task structure from the probe trials themselves.

We next turned to the fMRI data acquired during probe trials, which allowed us to test participants' ability to use conceptual knowledge to generalize under circumstances where the available information was incomplete (i.e., partial patterns; Figures 2D and 5), but did not provide opportunity for learning through feedback. We reasoned that if the hippocampus and vMPFC track the emergence of conceptual knowledge during learning trials, then these regions should also guide probe trial performance. To test this hypothesis, we performed a region of interest analysis in these two functionally defined regions, initially contrasting activity during the two varieties of probe trials, termed “outcome determined” and “outcome undetermined” (see Experimental Procedures; Figure 5). Critically, in outcome-determined trials, the outcome (i.e., sun/rain) could be accurately predicted based on conceptual knowledge of the task structure. In contrast, in outcome-undetermined trials, the outcome could not be predicted based on the information given (i.e., 50% sun, 50% rain), though these trials were otherwise closely matched to outcome-determined trials in terms of visual appearance and RT (outcome_determined 1.54 s, outcome_undetermined 1.58 s, p = 0.31).

Results from this analysis show that activity in the hippocampus, and vMPFC, was significantly greater in outcome_determined trials as compared to outcome_undetermined trials (functionally defined left hippocampus ROI: t = 1.83, p = 0.04; vMPFC ROI: t = 1.96, p = 0.03). Importantly, neural activity in these brain regions showed a significant correlation with performance in outcome_determined trials (hippocampus ROI: t = 1.87, p = 0.04; vMPFC ROI: t = 1.91, p = 0.03). Our findings, therefore, provide evidence that the hippocampus and vMPFC support neural representations of conceptual knowledge, which are used to guide participants' choices even in the absence of trial-by-trial feedback, when generalization is required because the exact situation has not been previously experienced during learning.

Taken together, our results show that neural activity in the hippocampus and vMPFC tracks the emergence of knowledge during the Initial session and its deployment during conceptual decision making. Previous work has emphasized the role of the vMPFC, and closely situated orbitofrontal cortex (OFC), in goal-directed decision making (Daw et al., 2005; Rangel et al., 2008; Rudebeck et al., 2008), based on outcome expectancies (Murray et al., 2007), simple if-then rules (e.g., match versus nonmatch [Hampton et al., 2006; Miller et al., 2002; Otto and Eichenbaum, 1992]), and the integration of social and reward information (Behrens et al., 2008). While conceptual knowledge has often been assumed to influence goal-directed behavior in humans (Shea et al., 2008), our study highlights its profound effect on participants' behavior and shows that this is reflected in neural activity in vMPFC, adding a further level of abstraction to the nature of neural representations it sustains.

#### New Session: Hippocampus Underpins Use of Conceptual Knowledge as a Guiding Schema in a Perceptually Novel Setting

**Figure 6:**
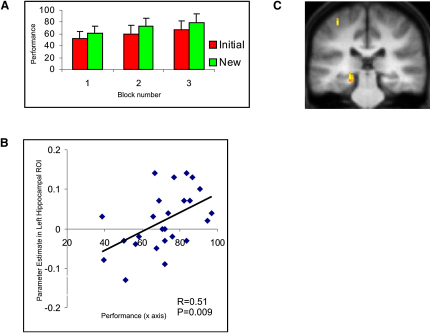
New Session: Behavioral and fMRI Data (A) Group-averaged (n = 25) performance (percent correct responses) in the first three blocks of the Initial session (red bars) plotted together with performance in the New session (green bars). Participants performed superiorly in the New session, as compared to the Initial session (t = 4.0, p < 0.001). Bars represent SEM. (B) Between-subjects correlation: the activity in left hippocampus of individual participants during learning trials in the Initial session correlated with their performance in New session (r = 0.51, p = 0.007), even once their performance in Initial session has been covaried out (p < 0.01). y axis: activity averaged across whole of left hippocampus during learning trials in the Initial session in arbitrary units (ROI analysis: see Experimental Procedures). x axis: performance (percent correct responses) averaged across three blocks of the New session. (C) Neural activity in the left posterior hippocampus exhibits a significantly stronger correlation with performance during learning trials in the New session as compared to the Initial session (x, y, z = −21, −30, −6; z = 3.40). Activation is significant at p < 0.001 uncorrected, and p < 0.05 SVC corrected. Overall network associated with proficient performance in the New session is illustrated in Figure S2 and detailed in Table S4. Activation is shown on the averaged structural MRI scan of the 25 participants, with color reflecting the z statistic (white > yellow > orange > red). Activation is shown at p < 0.005 for display purposes.

Having examined how conceptual knowledge is acquired during the Initial session, we next probed the neural mechanisms underpinning its application in a new setting (i.e., the New session), where, unbeknownst to the participants, the task structure was the same but the actual shapes novel. To successfully transfer previously acquired knowledge, participants were required to represent the higher-order task structure in an abstract form (i.e., not tied to individual shapes), reactivate this abstract conceptual representation (i.e., schema [Bartlett, 1932; Rumelhart, 1980]) appropriately in the New session, and use it to provide organizing principles to guide learning and choice behavior. That participants were able to do this is evidenced by their superior performance during the New session as compared to the Initial session (Figure 6A; t = 4.0, p < 0.001). While nonspecific skill learning effects are a well-recognized consideration in such circumstances, it is unlikely that they contribute significantly to the performance enhancement observed. Specifically, our neural findings argue strongly against this possibility (see below), as does the tight correlation observed at a behavioral level between the performance of an individual participant in the New session and the amount of conceptual knowledge acquired in the Initial session (r = 0.35, p = 0.04).

To discover the neural mechanisms responsible for this striking performance enhancement observed in the New session, we next turned to the fMRI data. We predicted that a brain region supporting schema representation and application should exhibit activity during learning trials in the Initial session which accounts for the considerable variability shown by individual participants in terms of performance enhancement in the New session. To test this hypothesis, we performed an ROI analysis in left hippocampus and vMPFC (see Experimental Procedures). Strikingly, activity averaged across the whole of the left hippocampus showed a significant correlation with performance in the New session (t = 2.7, p = 0.007; Figure 6B, see Experimental Procedures). Importantly, this correlation remained significant even once any effect of Initial session performance had been covaried out (p < 0.05), arguing against this finding representing a nonspecific effect associated with good performers in general. In contrast, activity in vMPFC, or indeed a PCC ROI, during the Initial session did not correlate with the performance of participants in the New session (both p > 0.1).

We also asked whether the correlation of hippocampal activity with New session performance reflects a nonspecific motor skill effect, indexed by the tendency of participants to be faster to respond in learning trials during the first block of the New session as compared to Initial session (Initial RT 1.62 s SD 0.15, New RT 1.43 s, SD 0.16; p < 0.05). No significant correlation was found between hippocampal activation and reduction in RT across sessions (r = 0.09, p > 0.1). Furthermore, the correlation of hippocampal activity with performance in the New session remained significant once both the effects of Initial session performance and the effect of RT speeding had been partialled out (r = 0.46, p = 0.02).

We next analyzed the fMRI data obtained from the New session in a similar way as previously (i.e., for the Initial session). Using a whole-brain analysis, we observed that activity in a network involving the hippocampus, vMPFC, and PCC showed a robust correlation with the probability of a correct response (i.e., probability_success) in the New session (Figure S2 and Table S4). The left posterior hippocampus alone (Figure 6C) showed a significantly stronger correlation with the probability of success on a given learning trial during the New session as compared to the Initial session (x, y, z = −21 −30 −6, z = 3.40). Interestingly, the region of left hippocampus identified in this analysis is more posterior to that observed in the Initial session. Previous studies have observed that activations within the hippocampus, and MTL, tend to be located toward its anterior aspect during encoding, and posterior during retrieval (Schacter and Wagner, 1999). As such, our observations are consistent with the notion that activation of the posterior hippocampus in the New session reflects schema retrieval/application, whereas activation of a more anterior region in the Initial session reflects schema formation. In marked contrast, differential activity in this analysis was not observed in vMPFC (p > 0.1), which tracked the probability of success in both New (Figure S2) and Initial sessions (Figure 3).

Taken together, these findings support a model in which the hippocampus and vMPFC interact during conceptual decision making but play dissociable roles. Specifically, our data, in linking hippocampal activity to subjects' ability to transfer knowledge to a novel setting, suggest that this region may house abstract conceptual representations of the task structure, which endure across time (e.g., across experimental sessions). In contrast, the vMPFC may act primarily to guide choices online, by integrating abstract information received from the hippocampus with stimulus-bound value information. As such, our results dovetail with recent perspectives arguing that the hippocampus plays a key role in decision making, by passing prospective memory signals coding for the available options to downstream valuation modules such as the vMPFC and OFC (Johnson et al., 2007). More generally, our findings accord with the recent demonstration that hippocampal amnesics, like patients with vMPFC damage, show decision-making impairments on the Iowa gambling task (Gupta et al., 2009).

### The Hippocampus, Conceptual Learning, and Semantic Memory

This study, in demonstrating that the hippocampus underpins the acquisition of conceptual knowledge, provides new insights into the types of neural representations and functions it support. While the hippocampus has been previously implicated in the representation of well-established concepts (e.g., Jennifer Aniston neurons [Quiroga et al., 2005]) and the generation of acquired equivalence between stimuli (Myers et al., 2003; Shohamy and Wagner, 2008), its role in knowledge acquisition, often subsumed within the broader notion of semantic memory, has remained controversial. In particular, it has been unclear whether patients with damage limited to the hippocampus show deficits in new semantic learning due to impaired episodic memory capacities (Tulving and Markowitsch, 1998), the role of the hippocampus as a “teacher” replaying individual memories to enhance knowledge acquisition in the neocortex (McClelland et al., 1995), or instead due to its function in the creation of linked networks of relational representations within a “memory space” (Cohen and Eichenbaum, 1993; Eichenbaum et al., 1999). Our results, in revealing how conceptual knowledge emerges through the abstraction of commonalities among multiple related experiences, provide empirical support for the latter memory space hypothesis of hippocampal function (Cohen and Eichenbaum, 1993; Eichenbaum et al., 1999). According to this perspective, therefore, the hippocampus supports conceptual learning through its unique associative memory capacities, which also explain its critical role in other relational memory domains (e.g., transitive inference paradigm [Preston et al., 2004]).

The current study also sheds new light on the nature of new semantic learning in amnesia. Previous work has shown that while patients with amnesia perform relatively well, or even normally (Duff et al., 2006), on certain tests of new semantic learning, information acquired tends to be rigidly organized (Bayley et al., 2008; Cohen and Eichenbaum, 1993; Duff et al., 2006; Eichenbaum, 2004; O'Kane et al., 2004; Westmacott and Moscovitch, 2001). Our findings suggest that a key deficit in such patients is the capacity to synthesize new concepts from related associative experiences, a function mediated by the hippocampus and critical in allowing knowledge to be flexibly accessed and deployed. In the future, it will be important to understand why patients with developmental amnesia, who have dysfunctional hippocampi, are able to achieve an apparently normal conceptual understanding of the world (Vargha-Khadem et al., 1997) and whether this arises as a consequence of neural plasticity resulting from early brain injury.

### Conclusions

While conceptual knowledge is thought to play an influential role in human decision making (Shea et al., 2008), the neural mechanisms underpinning its emergence and influence on choice behavior have been little studied until now. Here, we reveal that the vMPFC, in concert with the hippocampus, underpins conceptual decision making, implying that this neural circuit comprises an important, but until now neglected, part of the goal-directed system in humans. More generally, our findings, in elucidating the conditions under which the hippocampus and vMPFC are recruited into a simple choice scenario, offer a fresh perspective on the intriguing question of why these brain regions are engaged during such a diverse range of tasks (e.g., spatial navigation, imagination, autobiographical memory, self-projection, fear extinction [Bar, 2007; Buckner and Carroll, 2007; Hassabis and Maguire, 2007; Phelps et al., 2004]). We suggest, therefore, that this neural circuit may support a common core function during goal-directed cognition, regardless of whether it is oriented to the past, present, or future, whereby the vMPFC mediates the online integration and evaluation of associative information conveyed by the hippocampus.

## Experimental Procedures

### Participants

Twenty-seven healthy, right-handed native English speakers, who were currently undertaking or had recently completed a university degree, participated in this experiment (age range 19–31; 12 female). Two of these participants were excluded: one due to a keypad malfunction and the other due to consistently poor task performance (i.e., failure to exceed chance performance of 50% in either the Initial or New session). All participants gave informed written consent to participation in accordance with the local research ethics committee.

### Stimuli

Pictures of fractals, rather than real-life objects, were used in our experiment to investigate the learning of new concepts uncontaminated by previous experience outside the experimental context. Two sets of six fractal images were used during the main experiment: one set in the Initial session and one set in the New session. A separate set of fractals was used during a practice session prior to the experiment where participants were familiarized with the task. In each set, four fractals were presented during learning and probe trials, and a further two fractals were only ever seen during control trials (see below). Allocation of set was randomized across participants. Prior to each scanning session, participants briefly performed a simple one-back task where they viewed each individual fractal picture five times, in order to minimize stimulus novelty effects during scanning. Images were obtained from the internet (http://techrepublic.com.com/2346-10878_11-33277-7.html), resized, and placed on a square black background. Examples of fractals used in the experiment are shown in Figure 1.

### Task and Procedures

Participants were told to imagine themselves as a weather forecaster who has to predict if it will be sunny or rainy on the basis of a given “pattern” on the screen, which was said to represent constellations of stars in the night sky (Figure 1) (see Supplemental Instructions Document).

Participants' task was to learn how each of eight patterns (P1–P8), created from different combinations of four fractals (i.e., F1–F4), predicted the weather. Each pattern was made up of two fractal shapes: one in the center of the screen, and one either to the right or the left. As illustrated in Figure 1, two fractals (F1 and F2) were only presented on either the left- or right-hand side of the screen, with two fractals (i.e., F3 and F4) only appearing in the center of the screen. Since all eight patterns were constructed from the same four fractals, successful performance required participants to use associative information consisting of shape-location and shape-shape conjunctions rather than elemental information (e.g., single shape). Each pattern (e.g., P1) was associated with a given outcome (e.g., sun) with a 100% probability. This probability was fixed for the duration of the experiment and did not change. The eight patterns are illustrated with their outcomes (Figure 1). Note that this is an example, since the construction of patterns from fractals and the mapping of patterns to outcomes was changed between participants.

During *learning* trials, participants viewed a given pattern on the screen (e.g., P1), entered a prediction (e.g., sun), and received feedback regarding correctness (correct/incorrect) and reward (win/lose money) (Figure 2A and Supplemental Experimental Procedures). *Probe* trials, however, did not involve feedback and required participants to generalize, since they were confronted with partial patterns (i.e., “as if the sky was partially obscured by cloud”), therefore providing us with an online measure of the level of conceptual knowledge acquired throughout the experiment (Figures 2C and 5 and Supplemental Experimental Procedures). Participants were also asked to rate their confidence in their predictions during probe trials, by indicating whether they were “sure” or “not sure” by button press. Prior to the experiment, participants were instructed to select a sure confidence rating only if they felt “at least 90% certain” that their prediction was correct.

Scanning consisted of two main sessions, the Initial session (45 min) and the New session (15 min), which had the same higher-order task structure though differing in terms of the set of fractals used. At the start of the experiment, participants were told that they would take part in two sessions. However, they were not told what would happen during the second (New) session until after the completion of the first (Initial session).

The Initial and New sessions were composed of nine and three blocks, respectively. Each block was comprised of a 40 trial miniblock made up of 32 *learning* trials with 8 *control* (i.e., baseline: see Supplemental Experimental Procedures) trials pseudorandomly interspersed in between, followed by an eight trial miniblock of *probe* trials. The start of each miniblock was preceded with the relevant instruction (i.e., “Get ready for learning trials,” “Get ready for probe trials”). In the Initial session, participants were given a brief rest after every three blocks, though remained in the scanner. Participants were removed from the scanner after the Initial session, debriefed, and returned to the scanner after a short interval (5 min) for the New session. In total, therefore, the Initial session consisted of 288 learning trials, 72 control trials, and 72 probe trials, and the New session of 96 learning trials, 24 control trials, and 24 probe trials.

### Debriefing Protocol

Following the completion of each experimental session (Initial and New), participants were carefully debriefed in order to evaluate the presence and nature of conceptual knowledge concerning the task structure, and dissociate this from a more specific knowledge of outcomes associated with each pattern in isolation (see Supplemental Experimental Procedures and Questionnaire). Information obtained at debriefing was designed to provide a measure of explicit (i.e., consciously accessible) conceptual knowledge, indexing participants' ability to access and deploy conceptual knowledge in a context quite different from the original learning situation. As such, the composite debriefing score (see Supplemental Experimental Procedures) complemented online indices of conceptual knowledge acquisition (i.e., probe trial performance) obtained during task performance. Of note, our debriefing protocol, like probe trials, was carefully designed so as to avoid the provision of new information concerning the task structure to participants.

### Behavioral Analyses

Analyses were conducted using SPSS software (http://www.spss.com), Matlab 7.0 (http://www.mathworks.com/products/matlab), and using the state-space model toolbox obtained from http://www.neurostat.mit.edu.

To evaluate the correlation between performance on different patterns (i.e., P1–P8), we first extracted vectors coding for participants' responses in binary fashion (i.e., correct versus incorrect). We then computed correlation coefficients, as implemented in Matlab 7.0, between pairs of different patterns (e.g., P1 versus P2, P1 versus P3, etc.). For each individual participant, we calculated the average correlation coefficient between performance on learning trials *within* a given domain (i.e., spatial [P1–P4] and nonspatial [P5–P8]) and that *across* domains (e.g., P1–P5, P1–P6, etc.). We then asked whether, using a paired sample t test across the entire group, performance on learning trials within a domain showed a significantly greater correlation than across domains. This is what would be expected if participants integrated information across relevant patterns (e.g., P1–P4) rather than learning each pattern-outcome association (e.g., P1 = sun) in isolation.

### fMRI Design

The temporal pattern of stimulus presentation was designed to maximize statistical efficiency while preserving psychological validity, in line with established procedure (Frackowiak et al., 2004; Friston et al., 1998; Josephs and Henson, 1999). The trial onset asynchrony (TOA) for learning trials was 7 s (i.e., 5 s during which the pattern and outcome were presented followed by 2 s fixation cross). Given that the TOA is not a simple integer multiple of the TR (time for acquisition of one scanning volume = 4.05 s), trial onsets were automatically temporally jittered with respect to scan onsets (Frackowiak et al., 2004). Importantly, the haemodynamic response to events that occur a few seconds apart is explicitly modeled (via a haemodynamic response function) and therefore can be estimated separately for each event type by implementing the general linear model, as is standard when using statistical parametric mapping software (SPM5) (http://www.fil.ion.ucl.ac.uk/spm/) (also see below) (Friston et al., 1998).

### Imaging Parameters and Acquisition

T2-weighted echo planar images (EPI) with BOLD (blood-oxygen-level-dependent) contrast were acquired on a 1.5 tesla Siemens Sonata MRI scanner using a specialized sequence to minimize signal dropout in the medial temporal lobe (Deichmann et al., 2003). We used the following scanning parameters to achieve whole brain coverage: 45 oblique axial slices angled at 30° in the anterior-posterior axis, TR 4.05 s, 2 mm thickness (1 mm gap), TE 30 ms, in-plane resolution 3 × 3 mm, field-of-view 192 mm, 64 × 64 matrix. A preparation pulse (duration 1 ms, amplitude +1 mT/m^∗^ms) was used in the slice selection direction to compensate for through-plane susceptibility gradients predominant in the hippocampus (Weiskopf et al., 2005). High-resolution (1 × 1 × 1 mm) T1-weighted structural MRI scan were acquired for each participant after functional scanning. These were coregistered to the functional EPIs and averaged across participants to aid localization.

### fMRI Data Preprocessing

Images were analyzed in a standard manner using the statistical parametric mapping software SPM5 (http://www.fil.ion.ucl.ac.uk/spm/). After the first six “dummy volumes” were discarded to permit T1 relaxation, images were spatially realigned to the first volume of the first session, followed by spatial normalization to a standard EPI template, resulting in a functional voxel size of 3 × 3 × 3 mm. Normalized images were smoothed using a Gaussian kernel with full width at half maximum of 8 mm.

### fMRI Data Analysis

Following preprocessing, the event-related fMRI data were analyzed in SPM5 using the general linear model following established procedures (Frackowiak et al., 2004; Friston et al., 1998). We targeted our analyses to detect brain regions whose activation pattern during learning trials significantly correlated with participant-specific trial-by-trial regressors, namely the probability_success and probe_performance regressors.

#### Parametric Regressors

(1) Probability_success: for each individual subject, the state-space model was used to estimate learning curves for each pattern (i.e., P1–P8) (see Supplemental Experimental Procedures). These learning curves constituted vectors indexing the probability of a correct response on a given trial and were used to create participant-specific parametric regressors.
(2) Probe_performance: probe trial performance was scored in accordance with the instructions given to participants about how to rate their confidence in their predictions. Correct predictions that were given a sure confidence rating were scored more highly than those accorded a not-sure rating, with the former scoring 5 points and the latter 2 points. Performance on spatial and nonspatial outcome_determined probe trials was then used to modulate respective learning trials (i.e., spatial: P1–P4/nonspatial: P5–P8) during the preceding miniblock. For instance, performance on the spatial outcome_determined probe trial illustrated in Figure 2C (upper panel) was used to modulate the four learning trials during the preceding miniblock where patterns P1 and P3 were presented. Similarly, performance on the nonspatial outcome_determined probe trial illustrated in Figure 2C (lower panel) was used to modulate the four learning trials during the preceding miniblock where patterns P7 and P8 were presented.

#### Specification of First-Level Design Matrix and Model Estimation

As a first step, the 5 s period during which pattern and outcome were displayed during learning trials was modeled as a boxcar function and convolved with the canonical haemodynamic response function (HRF) to create regressors of interest. In initial analyses, vectors indexing spatial (i.e., P1–P4) and nonspatial (i.e., P5–P8) learning trials were coded separately in the design matrix. Given that no significant effects of domain (i.e., spatial versus nonspatial) were found, even at liberal statistical thresholds (p < 0.01 uncorrected) (see Supplemental Results), spatial and nonspatial trials were included within a single regressor in all subsequent analyses.

Participant-specific vectors coding for probability_success, probe_performance were then included as parametric modulators in the design matrix. In the first analysis reported, only probability_success was included as a parametric regressor. In subsequent analyses set up to identify brain regions whose activity specifically tracks the emergence of conceptual knowledge, we included the probe_performance vector as an additional (second) parametric regressor in the design matrix. Of note, the correlation between these two regressors before inclusion in the first level design matrix was ∼0.42 across subjects. After the automatic orthogonalization procedure implemented in SPM5, the correlation between these two regressors averaged across subjects was −0.08.

These parametric regressors were also convolved with the HRF, leading to the height of the HRF for a given event being modulated as a function of the probability of success, or probe performance. Thus, these regressors model BOLD signal changes that covary with probability of success, or probe performance on a given trial. We also included vectors coding for outcome_determined, and outcome_undetermined, probe trials, and control trials, in the first level design matrix. Probe performance was also included as a parametric regressor relating to neural activity during probe trials. Further, participant-specific movement parameters were included as regressors of no interest. A high-pass filter with a cutoff of 180 s was employed. Temporal autocorrelation was modeled using an AR(1) process.

Model estimation proceeded in two stages. In the first stage, condition-specific experimental effects (parameter estimates, or regression coefficients, pertaining to the height of the canonical HRF) were obtained via the GLM in a voxel-wise manner for each participant. In the second (random-effects) stage, participant-specific linear contrasts of these parameter estimates, collapsed across the three sessions, were entered into a series of one-sample t tests (as is standard when using SPM and a factorial design [Frackowiak et al., 2004]), each constituting a group-level statistical parametric map.

### Statistical Inference

#### Voxel-Based Analyses

We report results in a priori regions of interest (previously defined in neuroimaging studies of decision making and associative learning [Hampton et al., 2006; Law et al., 2005; O'Doherty, 2004]: MTL, vMPFC, PCC, amygdala) where activations are significant at p < 0.001 uncorrected for multiple comparisons with an extent threshold of 5 voxels, and survive small volume correction (SVC) for multiple comparisons (or family-wise error [FWE] correction across the whole brain). The SVC procedure, as implemented in SPM5 using the FWE correction procedure (p < 0.05), allows results to be corrected for multiple nonindependent comparisons with a defined region of interest. For the SVC procedure, we used an anatomical masks obtained from the MarsBar SPM toolbox (hippocampus, amygdala) (http://marsbar.sourceforge.net/), and a 4 mm sphere centered on coordinates derived from previous work (vMPFC: x, y, z = 6 57 −6 [Hampton et al., 2006]). Activations in other brain regions were only considered significant if they survived whole-brain FWE correction for multiple comparisons at p < 0.05 (in line with established procedures [Frackowiak et al., 2004]), but are reported for completeness at a threshold of p < 0.001 uncorrected for multiple comparisons. All activations are displayed on sections of the average structural image of all the participants. Reported voxels conform to Montreal Neurological Institute (MNI) coordinate space. Right side of the brain is displayed on the right side.

#### Region of Interest Analyses

To test whether brain regions, namely the left hippocampus and vMPFC, which track the emergence of conceptual knowledge also guide probe trial performance, we performed an ROI analysis in these two functionally defined regions (using the MarsBar SPM toolbox: http://marsbar.sourceforge.net/). These regions were functionally defined from the group statistical map pertaining to the correlation of brain activation with probe_performance and thresholded at p < 0.005 uncorrected. Thus, definition of this ROI is unbiased with respect to our contrast of interest, which pertains to neural activity during probe trials. Using the MarsBar SPM toolbox, we obtained parameter estimates for all voxels within this region, for the group as a whole. These parameter estimates were averaged across the ROI and specific effects tested by one-sample t tests.

We also performed an ROI analysis to ask whether activity within the hippocampus during learning trials in the Initial session accounts for the considerable variability shown by individual participants in terms of performance enhancement in the New session. Here, we used an anatomically defined mask of the (left) hippocampus obtained from the MarsBar SPM toolbox (http://marsbar.sourceforge.net/). This analysis was implemented using the multiple regression function in SPM5, with the effect of performance in the Initial session included as a covariate of no interest.

It is important to note that these analyses treat data from a ROI as if it was from a single voxel, and hence no correction for multiple comparisons is necessary.

#### Psychophysiological Interaction Analysis

A PPI analysis is employed to identify the presence of functional coupling between different brain regions, by showing that activity in a distant region can be accounted for by an interaction between the influence of a source region and an experimental parameter (Friston et al., 1997). We used a PPI analysis to ask whether the left hippocampus, our source region, significantly influenced activity in vMPFC (or PCC), specifically in relation to the level of conceptual knowledge acquired (i.e., condition × probe_performance interaction). To do this, we used SPM5 to first extract the time series for the peak voxel in the left hippocampus (i.e., 4 mm sphere centered on peak coordinate in the group analysis x, y, z, = −24, −25, −21), identified in the correlation of learning trial related activity with probe_performance (Figure 4) (physiological effect). This time course was the first regressor in the PPI analysis. Next, we calculated the product of the time course and the probe_performance vector to create the PPI (i.e., psychophysiological interaction) term. The effect of this interaction term was assessed for each participant and entered into a second level group-level analysis. Specifically, we performed an ROI analysis in the functionally defined region of the vMPFC (see above) to ask whether this region shows significant functional coupling with the left hippocampus, the magnitude of which specifically tracks the amount of conceptual knowledge deployed during learning trials.
