# Supplementary material for: Tracking the Emergence of Conceptual Knowledge during Human Decision Making
Source: Neuron. 2009 Sep 24;63(6):889–901. doi: 10.1016/j.neuron.2009.07.030 (PMC2791172; doi:10.1016/j.neuron.2009.07.030)
Supplement: Document S1. Supplemental Data [file mmc1.pdf]

Neuron, Volume 63

Tracking the Emergence of Conceptual Knowledge during Human Decision Making

Dharshan Kumaran, Jennifer J. Summerfield, Demis Hassabis & Eleanor A. Maguire

SUPPLEMENTAL MATERIAL

Supplemental Results

Supplementary Behavioral Experiment

Thirteen participants (average age 25 (range 19-30), 5 male; written consent obtained in line with guidelines of local ethics committee) took part in this experiment, which was conducted primarily to examine the relationship between probe trial performance and participants' ability to verbally express the underlying conceptual structure of the weather prediction task. The experimental design was directly analogous to that used in the fMRI experiment, except that measures of verbally expressed conceptual knowledge were obtained after each block of learning trials, rather than solely at the end of the session as in the fMRI experiment (see Supplemental Experimental Procedures).

Average learning trial (behavioral expt: mean 74.9%, SD 9.1; fMRI experiment: 73.1% , SD 13.8) and probe trial performance (behavioral expt: mean 2.8, SD 1.1; fMRI experiment: 2.6, SD 0.8) in this experiment were similar to that of the main fMRI experiment (both $p>0.1$). This suggests that participants performed the task in a similar fashion in both experiments, despite minor differences between experimental

designs. Participants ability to verbally state the conceptual structure of the task increased from close to zero following the end of the first learning block of the Initial session (mean 0.6, SD 1.5) to a mean of 13.1 points (SD 8.5) following the last block (maximum score 20 points: see Supplemental Experimental Procedures). Critically, a robust correlation was found between probe performance and verbal task structure description score ($r=0.71$, $p<0.001$), which remained significant after the effect of learning trial performance had been partialled out ($r=0.55$, $p<0.001$). By linking probe performance to verbally expressed conceptual knowledge in this way, this finding provides further evidence consistent with the notion that probe trial performance involves the use of conceptual knowledge.

FMRI analyses (Initial Session): spatial vs non-spatial

At a behavioral level, performance was well matched during spatial and non-spatial learning (RT: spatial 1.37 sec, non-spatial 1.36 sec, $p=0.8$; performance: spatial=74.9%, non-spatial=75.0%, $p=0.9$) and probe (performance: spatial 2.3, non-spatial 2.7, $p=0.25$) trials. We conducted initial analyses to identify brain regions whose activity during learning trials differed as a function of domain (i.e. spatial vs non-spatial). No significant activation was observed, even at liberal statistical thresholds ($p<0.01$ uncorrected), in a priori regions of interest, including the hippocampus. Whilst this result is a null finding and therefore should be interpreted with caution, it is consistent with our previous finding that the hippocampus is critical for both non-spatial and spatial learning in this task (Kumaran et al., 2007), and theoretical perspectives relating to the role of the hippocampus in domain-general relational learning (Cohen and Eichenbaum, 1993; Mayes et al., 2007; McClelland et al., 1995).

FMRI analyses (Initial Session): supplemental analyses for correlation with probability_success

Firstly, we included reaction times for each trial as a covariate of no interest to discount the possibility that this brain network is activated because participants have more time to engage in unconstrained cognitive activity (e.g. daydreaming) as their performance becomes more fluent. Inclusion of this reaction time regressor in the model had no significant effect on the pattern of activations observed in relation to the probability_success regressor. Secondly, we considered the possibility that activation in this network reflects the provision of correct feedback itself, rather than the probability of a correct response. To address this, we conducted an analysis where correct trials were modelled separately from incorrect trials. This verified that activation of this network was related to the probability of success, rather than provision of correct (or incorrect) feedback alone, since we observed a qualitatively similar network to be active when only correct trials were considered. Finally, we considered the possibility that the correlation of activity in vMPFC and other areas with probability of success may arise due to a non-specific scanner drift. We discounted this by showing that a similar pattern of activation is observed when one searches for voxels showing a significantly greater correlation with probability of success, as compared to a linear time-dependent drift in a no-learning control condition.

It is worth noting that we observed activation in a network involving dorsolateral PFC, insula, anterior cingulate cortex, intraparietal sulcus when we searched for areas whose activity *inversely* correlated with the probability of success (Figure S1, Table S3). The reaction time regressor, however, when included as a covariate of no

interest, accounted for a large proportion of activity in this network, suggesting engagement of this network may reflect increased attentional demands or time-on-task.

Supplemental Experimental Procedures

Task

Learning trials: The timeline of a learning trial is shown in Figure 2A. Each trial began when two shapes were presented on the screen. Presentation of all 8 patterns (i.e. P1-8) was pseudorandomly intermixed, with each pattern presented 4 times during each miniblock (i.e. 36 times in total during the Initial session). Participants were told to enter their prediction (i.e. Sun or Rain) as soon as they were ready on a 4 button MR compatible keypad, using the index or middle finger of their right hand. After a 3 second period, the screen advanced to display a graphical representation of the outcome for that trial (e.g. picture of sun: Figure 2A), with text informing participants whether they had made a correct or incorrect response (e.g. "correct"), and whether they had won or lost money (i.e. green or red money bag). The screen indicating the trial outcome was displayed for 2 seconds following which a fixation cross was displayed for 2 seconds. After this, the next (learning or control) trial began. Participants were told that they would receive a minimum of £15, and would be paid £0.10 for a correct prediction, and would lose £0.10 for an incorrect prediction. Hence, performances of 90%, 75%, and 50% correct predictions for the entire experiment would yield £50, £36, and £15 respectively.

Critically, whilst participants could simply learn the correct response associated with a given pattern in isolation (e.g. pattern 1 – Sun, pattern 2 - Rain), there was also the opportunity for them to acquire spatial and non-spatial conceptual knowledge, which was assessed in probe trials (Figures 2C, 5) at the end of each learning block and using a debriefing protocol. Specifically, participants could learn that fractal 1 predicts sun when on the left, and rain when on the right, irrespective of the identity

of the central shape (i.e. fractal 3 or 4), by abstracting the commonalities across the relevant patterns, therefore termed "spatial" (i.e.P1-P4)(Figure 1). In a similar vein, participants could learn that the shape-shape combination of fractals 2 and 3 predicts sun, and 2 and 4 rain, regardless of the position of fractal 2, by appreciating the relationship between the relevant "non-spatial" patterns (i.e. P5-8).

Probe trials were included to provide an online measure of the amount of spatial and non-spatial conceptual knowledge acquired. During these trials, participants were required to enter a weather prediction based on partial patterns (i.e. "as if the sky was partially obscured by cloud"), rather than complete patterns as presented in learning trials. Whilst participants were informed prior to the experiment that they would still be rewarded at the end of the experiment for correct predictions on probe trials, they were not issued with trial-by-trial feedback to prevent learning in probe trials.

Spatial and non-spatial probe trials were pseudorandomly intermixed during probe trial miniblocks. In spatial probe trials (Figure 2C upper panel), one fractal (i.e. either F1 (illustrated) or F2) was presented on either the left (illustrated) or the right. A question mark displayed in the central position indicated that the identity of the central fractal was not known. In non-spatial probe trials (Figure 2C lower panel), one fractal was presented in the central position (F3, or F4 (illustrated)), with another above (F1 or F2 (illustrated)). The question mark indicated that the position of the peripheral fractal was not known.

Importantly, we included two varieties of probe trials, termed "outcome-determined" and "outcome-undetermined", which allowed us to assess participants' conceptual

knowledge, without giving them new information about the underlying task structure (Figure 5). In outcome-determined trials, the main trials of interest, participants could deploy conceptual knowledge to make accurate predictions: specifically, in spatial probe trials of this type the presence of F1 on the left is predictive of Sun, regardless of the identity of the central shape (shown in Figure 5). In non-spatial outcome_determined probe trials, the presence of fractals F2 and F4 on the screen (shown in Figure 5) is indicative of rain, regardless of the position of F2.

In contrast, conceptual knowledge could not be deployed in outcome-undetermined trials where the outcome could not be predicted based on the information given (i.e. 50% S, 50% R). These trials, however, were otherwise closely matched to outcome-determined trials, providing an appropriate comparison condition for the neuroimaging analyses, as well as serving the function of preventing participants from gaining information about the higher order task structure from the probe trials themselves.

Participants were also asked to rate their confidence in their predictions during probe trials, by indicating whether they were “sure” or “not sure” by button press. Prior to the experiment, participants were instructed to select a sure confidence rating only if they felt “at least 90% certain” that their prediction was correct. That participants adhered to this criterion is attested by the very few numbers of sure confidence ratings accorded to predictions in outcome-undetermined trials (9% confidence ratings in outcome_undetermined vs 41% in outcome_determined trials), where the outcome could not be predicted based on the information provided.

Control trials: These trials were designed to be similar to learning trials in terms of visual display, requirement for motor response, reward (i.e. presence of positive and negative monetary feedback), but without requiring participants to learn the outcome associated with a given pattern. As such, control trials were included to provide a baseline control for the fMRI analyses and were pseudorandomly intermixed with learning trials. Within a session the same two fractals (F5, F6) always appeared during control trials. One fractal (e.g. F5) appeared always on either the right or left of the screen, and the other (e.g. F6) in the centre.

The timeline of control trials was analogous to learning trials. However, an asterisk appeared below one of the fractals indicating to the participants which button (i.e. index of middle finger) they should depress. Participants were advised, however, that the outcome would be determined randomly, and therefore that over the course of the experiment they would, on average, not gain or lose money on these trials.

Debriefing Protocol

Participants were first asked an open-ended question requiring them to spontaneously describe how they had performed the task, and to state how they thought outcomes were related to shape configurations (i.e. the underlying task structure). Pictures of the individual fractals were made available to the participants to aid their description. This was scored (max score=4) as follows: 1 point was awarded for each component of conceptual knowledge that participants correctly stated (e.g. "This shape (participants points to picture of F1) on left means Sun regardless of what the central shape is)). 0 points were awarded for incorrect or incomplete statements.

Participants were next asked to estimate outcome probabilities in a given situation. Importantly, participants were not queried about specific patterns that they had experienced (e.g. P1) but about more general situations (e.g. fractal F1 left, regardless of fractal in the centre: see see Supplemental Debriefing Protocol). This was done in order to assess participants' conceptual knowledge of the underlying task structure.

The test was scored as follows: a participant's estimated probabilities were compared to the correct answers (i.e. true probabilities) for each question. The average deviation of participants' answers from the true probabilities was calculated across all the eight questions (i.e. 0% if all answers correct). We determined a score that if a participant was answering randomly (i.e. at chance level), then his/her answer to each question would on average be 50%. The true probabilities for each question are either 0% (e.g. how often is fractal F1 (picture shown to participant) on the right associated with a sunny outcome?), 50% (e.g. how often is a fractal F2 on the left associated with a sunny outcome?), or 100% (e.g. how often is a square on the left associated with a sunny outcome?). It therefore follows that the score expected by chance in this test is an average deviation of 25% from the true probabilities. In this way, we were able to determine if the average deviation for each participant from the correct probabilities was significantly different to that expected by chance.

A composite score was calculated to index participants' level of explicit conceptual knowledge demonstrated at debriefing. This was done by transforming scores from participants' spontaneous description of the task structure and their probability estimates to z-scores and then averaging them.

Estimation of learning curves

State-space model. Analyses were conducted using the state-space model toolbox obtained from www.neurostat.mit.edu. The state-space model is a dynamic estimation method, which computes an estimate of the learning curve (i.e. probability of correct response as a function of trial number) by maximising the likelihood of the observed data (i.e. sequence of binary responses), using an expectation maximisation (EM) algorithm (Smith et al., 2004; Wirth et al., 2003).

Whilst the state-space model is described in detail and validated in Smith et al. (Smith et al., 2004), a summary is presented here. The state-space model involves two equations: a state equation and an observation equation. The state equation defines a hidden (i.e. unobservable) learning process, which evolves across trials through a Gaussian random-walk model. Importantly, the state-space smoothing algorithm employed here conducts analyses from the perspective of an ideal observer, where the learning state process at each trial is estimated after seeing the outcomes of all trials. As such, the state-space model differs from analytic methods such as reinforcement learning (RL) algorithms (see below)(e.g. (Watkins and Dayan, 1992)) which view learning from the perspective of the subject carrying out the task, where estimates of the learning process are based on past data up to the current trial. The observation equation, expressed as a Bernouilli probability mass function, completes the state-space model setup and defines how the observed binary performance data relate to the hidden learning process.

The state-space model is defined as follows: assuming there are k trials of a given pattern (e.g. P1 in the current experiment), the probability of observing a response on

a given trial (n_k) that is correct (i.e. $n_k = 1$) is governed by a hidden learning process x_k which changes as a function of trial number. The observation model, expressed as a Bernoulli probability mass function, defines the probability of observing n_k (i.e. either correct=1, or incorrect=0) given the value of x_k.

$$Pr(n_k|p_k, x_k) = p_k^{nk}(1- p_k)^{1-nk}$$

p_k is defined by the following logistic equation, which ensures that at each trial the probability of a correct response is constrained between 0 and 1. μ is a constant defined by the probability of a correct response in the absence of learning (i.e. $p_0 = 0.5$ in the current experiment):

$$p_k = \exp(\mu+x_k)/(1+\exp(\mu+x_k))$$

The hidden learning process is defined as a random walk, where k is the number of trials in the experiment, and ε_k are independent Gaussian random variables with mean 0 and variance σ_e^2 .

$$x_k = x_{k-1} + \varepsilon_k$$

The objective of the analysis is to estimate p_k (i.e. probability of correct response) for k=1, 2…..k (i.e. for each trial) by computing estimates of $x = \{ x_{0,} x_{1,} x_{2,}…….. x_k \}$ and σ_e^2 , using an EM algorithm. The EM algorithm is a widely used procedure for performing maximum likelihood estimation when there is an unobservable process.

For details of the specific EM algorithm used, including filter and fixed interval smoothing algorithms, see Appendix A of Smith et al (2004).

Reinforcement learning model. We used a standard RL algorithm, termed Q-learning (Watkins and Dayan, 1992), to derive estimated learning curves that best fit the binary performance data. In a learning trial in our task, participants were presented with a pattern (e.g. P1) and had to choose between two actions, one of which was correct and rewarded (e.g. button coding sun: +£0.10), and the other incorrect and penalized (e.g. button coding rain: - £0.10). The RL model we used updates the values of actions using the Rescorla-Wagner learning rule (Rescorla and Wagner, 1972):

$$V(a)_{t+1}= V(a)_t +\eta\delta_t$$

Where η is the learning rate, $V(a)_t$ is the value of action *a* at trial number *t*. δ_t is the prediction error at trial *t*, and is the difference between expected and observed outcome:

$$\delta_t=R(t)-V(a)_t$$

Where R(t) is the actual reward on trial *t*, which could be £0.10 or -£0.10 in our experiment. Given these calculated action values, the probability of selecting each action was estimated using the a standard stochastic decision principle, the softmax rule:

$$P(a)_t=e(V(a)_t/\beta)/(e(V(a)_t/\beta)+ e(V(b)_t/\beta))$$

Where $P(a)_t$ is the probability of choosing action *a* at trial *t* and β is the temperature coefficient.

To compute the parameters of the RL model which best fit the binary performance data, we used optimization functions implemented in Matlab 7.0. These adjust the value of the η (learning rate) and β (temperature) parameters to maximise the likelihood of the observed data under the model. In this way, we determined the best-fitting RL model across the entire group of 25 subjects, and for each subject or pattern individually.

Moving average method. This technique, described fully in Smith et al. (Smith et al., 2004), has often been used for estimating a learning curve (i.e. the probability of a correct response at trial *t*). It does this by calculating average performance within a series of overlapping windows of length 2*w*+1,

$$P_t = (2w+1)^{-1} \sum_{t-w}^{t+w} n_t$$

where P_t is the probability of a correct response at trial *t*, n_t is the outcome at trial *t* (i.e. 1 if correct, and 0 if incorrect), and *w* in our experiment was set at 3, giving a window size of 7 trials. To calculate P_t for trials at the start and end of the experiment, a forward-only or backward-only window is used.

Model Fitting

Model fitting was carried out by comparing the series of binary responses generated for each of the three models (i.e. state-space model, RL model, moving average method), with participants' actual binary performance data. For each subject, a mean squared error (MSE) term was computed for each model by averaging across all patterns (i.e. P1-P8), in line with standard procedures. To verify that the state-space model provided a better fit to the binary choice data observed in our experiment, we performed comparisons with a standard RL model (Q-learning (Watkins and Dayan, 1992)) and the moving average method (see Supplemental Experimental Procedures: (Smith et al., 2004)). In all comparisons performed, learning curves produced by the state-space model provided the best fit to the observed data. As such, the state-space model outperformed the RL model, both when the parameters of the RL model were optimized over the entire group of subjects (α=0.06, β=0.20), and when parameters were optimized to individual subjects or patterns. Average MSE per subject for each model, in arbitrary units, were: 639 (SD 472) for the state-space model, 913 (SD 654) for the moving average method, and 999 (SD 670) for the best-fitting RL model. As such, across the entire group, the fit of learning curves generated by the moving average method was intermediate: i.e. worse than the state-space model, but superior to the best-fitting RL model. State-space model generated learning curves were therefore used as parametric regressors in all fMRI analyses reported (see below). Of note, we also carried out analogous analyses using learning curves generated using the moving average method. These results, for both the Initial and New sessions were qualitatively very similar to the reported analyses.

Supplementary Behavioral Experiment

The experimental design and timing of stimulus presentation was identical to that used in the fMRI experiment with the following exceptions: (1) After each learning trial block, participants were asked an open-ended question requiring them to verbally express "anything that they had been using to predict the weather". As during probe trials, participants were asked to provide confidence ratings on their verbal statements (guess/fairly sure/very sure). Pictures of the individual fractals were made available to the participants to aid their description. No feedback was given, and no additional questions were asked. This was scored (max score=4) in the same way as the debriefing reports from the fMRI experiment (see Experimental Procedures): 1 point was awarded for each component of conceptual knowledge that participants correctly stated (e.g. "This shape (participants points to picture of F1) on left means Sun regardless of what the central shape is")). 0 points were awarded for incorrect or incomplete statements. As for probe trials, scoring of verbal statements was weighted similarly according to confidence rating (see below), yielding an overall maximum score of 20 points. In this way we obtained on-line measures of participants' ability to state the conceptual structure of the task, which was used in correlational analyses with probe performance. (2) Each probe trial block consisted of 16 trials, rather than 8 trials as used in the fMRI experiment. (3) Participants rated their confidence in their probe trial predictions according to guess ("little idea of correct response or completely guessing")/fairly sure (i.e. "around 70% sure") / very sure ("around 90% sure"). As in the fMRI experiment probe trial performance was scored in accordance with the instructions given to participants about how to rate their confidence in their predictions: correct predictions that were given a very sure confidence rating were scored at 5 points, those given a fairly sure rating 3.5 points, and guess response 2

points. (4) The Initial session comprised 6 learning blocks (as compared to 9 blocks in the fMRI experiment), and the New session 3 learning blocks. (5) The baseline condition consisted of 2 different fractals (i.e. F5,F6) which were linked to the outcome (i.e. sun/rain) with 100% contingency, rather than being random (i.e. 50% sun, 50% rain) as in the fMRI experiment.

Supplemental Table 1. Brain areas whose activity significantly correlated with the probability of a correct response on a given learning trial (i.e. probability_success) during the Initial session.

Region	Laterality	x	y	z	z-score
vMPFC	L	-9	45	-9	5.60**
		-3	57	0	5.53**
Parahippocampal cortex	L	-27	-24	-18	5.60**
(extending into hippocampus		-24	-36	-12	3.7
bilaterally)	R	27	-18	-18	4.6
		36	-27	-15	3.9
PCC	L/R	0	-30	39	5.08**
(extending into retrosplenial cortex)	R	3	-18	57	4.73
Amygdala	L	-15	0	-15	4.99**
Ventral Striatum	L	-15	6	-9	4.57
	R	21	12	-9	3.67
Angular gyrus	L	-50	-70	33	4.43
Middle temporal gyrus	R	63	-9	-18	3.62
Inferior temporal gyrus	L	-54	-15	-27	3.43
Caudate (head)	L	-18	15	9	3.56

All regions are significant at p<0.001 uncorrected for multiple comparisons, with an extent threshold of 5 voxels. All coordinates are in MNI space.
** indicates survives FWE correction procedure across whole brain.

Supplemental Table 2. Brain areas whose activity significantly correlated with performance on probe trials (i.e. probe_performance) during the Initial session.

Region	Laterality	x	y	z	z-score
vMPFC	L	-3	51	-15	4.94**
		-9	45	3	4.88**
Hippocampus	L	-24	-15	-21	3.93
PCC	L	-3	-48	33	3.61
Angular Gyrus	L	-51	-69	27	3.71

All regions are significant at p<0.001 uncorrected for multiple comparisons, with an extent threshold of 5 voxels.
** indicates survives FWE correction procedure across whole brain.

Supplemental Table 3. Brain areas whose activity was *inversely* correlated with the probability of a correct response on a given learning trial (i.e. probability_success) during the Initial session.

Region	Laterality	x	y	z	z-score
Insula	L	-33	15	3	5.33**
	R	33	24	3	4.65
Cerebellum	L	-21	60	33	4.80*
Dorsolateral PFC	L	-42	27	21	4.00
	R	54	27	33	4.36
Supplementary motor area/ Anterior cingulate cortex	L	-6	18	45	3.99
Ventrolateral PFC	R	39	60	6	3.57
	L	-36	54	3	3.41
Inferior occipital gyrus	L	-39	-60	12	3.61

All regions are significant at $p<0.001$ uncorrected for multiple comparisons, with an extent threshold of 5 voxels.
** indicates survives FWE correction procedure across whole brain.

Supplemental Table 4. Brain areas whose activity significantly correlated with the probability of a correct response on a given learning trial (i.e. probability_success) during the New session.

Region	Laterality	x	y	z	z-score
vMPFC	L	-3	42	-9	5.07**
Hippocampus (posterior) (extending into parahippocampal cortex)	L	-21	-30	-6	4.90**
	R	18	-30	-9	3.64
Hippocampus (body)	L	-24	-18	-15	3.73
	R	21	-18	-18	4.55
PCC	R	6	-42	30	3.95
Amygdala	L	-24	-3	-18	3.33
Angular gyrus	L	-18	-42	66	4.22
Caudate (head)	R	18	18	12	3.51

All regions are significant at p<0.001 uncorrected for multiple comparisons, with an extent threshold of 5 voxels.
** indicates survives FWE correction procedure across whole brain.

Figure S1. Brain areas *inversely* correlated with the probability of a correct response in the Initial session.

Brain areas whose activity during learning trials shows a significant *negative* correlation with a participant-specific index of performance (probability_success). Activations are shown on the averaged structural MRI scan of the 25 participants, with color bar indicating the t-statistic associated with each voxel and the z-score equivalent. Top panel shows activation in supplementary motor area/anterior cingulate cortex. Lower panel shows activation in bilateral insula, bilateral dorsolateral PFC, and anterior cingulate cortex. See Supplemental Table 3 for a full list of activations. Activations were significant at $p<0.001$ uncorrected, shown at $p<0.005$ (uncorrected) for display purposes.

Figure S2. Brain areas associated with proficient performance during learning trials in New session.

Brain areas whose activity during learning trials shows a significant positive correlation with a participant-specific index of performance (probability_success). Activations shown on the averaged structural MRI scan of the 25 participants, with color bar indicating the t-statistic associated with each voxel and the z-score equivalent. Upper left panel shows activation in bilateral posterior hippocampus extending into left parahippocampal cortex. Activation in PCC is also displayed. Upper right panel shows activation throughout left hippocampus. Lower left panel shows activation in vMPFC, bilateral hippocampus extending into left amygdala and left parahippocampal cortex. See Supplemental Table 4 for a full list of activations. Activations in left posterior hippocampus and vMPFC were significant at $p<0.05$

FWE corrected (see Experimental Procedures). Activations are shown at $p<0.005$ (uncorrected) for display purposes.

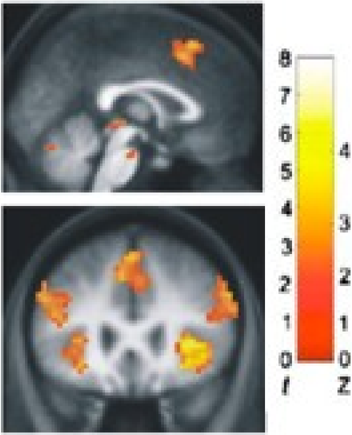

FIGURE S1

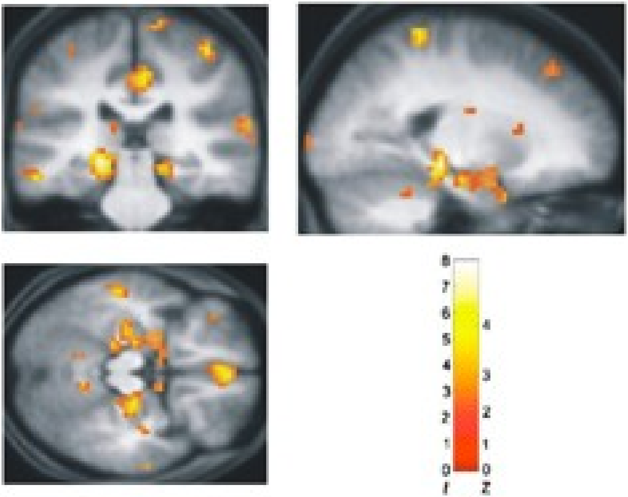

FIGURE S2

Instructions for the Weather Prediction Task

Initial Session

Imagine you are a weather forecaster who is trying to predict whether it will be SUNNY or RAINY on the following day. You make this prediction based on the shapes that constellations of stars form in the night sky.

So the way it works is like this (using a computerized demo): imagine you are looking at the sky and ahead of you see this shape, and to your left you see this shape. What you need to do is to predict whether it will be Sunny/Rainy on following day using these keys. You will be given a limited time in the real experiment. Then you will be shown the outcome, together with correct/incorrect. If you are correct you will gain 10p and if incorrect lose 10p. If you perform at 90%, you will get £50, if you perform at 75%, you will get £36, and if you perform at 50% (i.e. guess) you will get £15. This is why it is important to perform well until the end - we want you to win as much money as possible.

I am not allowed to tell you how this information can be used to predict whether it will be sunny/rainy but what I can tell you is that: Firstly, it is possible to accurately predict the weather based on the shapes you see. Secondly, you will see the same 4 shapes throughout the experiment. Thirdly, the way that shapes predict the weather will not change over the experiment so once you learn it, you should be able to keep getting it right. Obviously at the start, you will have to guess what the weather will be like, but the idea is that you learn how to predict the weather as the experiment unfolds. But don't worry if at any stage you are not sure what prediction to make - to

guess is better than not to answer at all (since you will be marked wrong for no answer).

You will also see “probe trials” which are a bit different. Here you are provided with only limited information about what the night sky looks like- as if it is partially obscured by cloud. In this type of probe trial, you know that this shape is in the left position but you don’t know which of the two shapes is in the centre. You are asked to predict whether based on this limited information it will be sunny or rainy tomorrow. So say you say sun, then will see this screen asking you whether sure or not. Answer with L and R buttons as shown. You will then be asked whether you are “sure” or “not sure”. Only answer “sure” if you are 90% confident in your prediction. In probe trials, you won’t be shown the outcome here, but you will be still paid according to your performance. In this type of probe trial, you know that this shape is in the centre, and this shape is on the side, but you don’t know which side it is on. Again you enter a prediction etc.

Importantly, in some probe trials, you will be able to make a correct prediction based on this limited information, but in others it won’t be able to make an accurate prediction based on what you are told i.e. it could be either sun/rain. In this case, just make a choice and choose “not sure” later.

You may find these probe trials difficult to start with; just do your best.

These are what we call “baseline” trials. You will see two other shapes that you won’t see in other trials. Asterix is telling you which button to press. If the asterix indicates

leftmost shape, then press the left button (e.g. sun). You will then see the outcome, which will be random (i.e. 50% rain and 50% sun).

New Session

*Note: Participants were only instructed about the New Session after the Initial session was over.

They were told: the instructions for this session are exactly the same as before, and you need to learn how to predict the weather. You’ll be doing this with a different set of shapes though.

Good Luck!

Debriefing Protocol for the Weather Prediction Task

General Questions

- How did you find that?
- Can you say briefly what the task was about, and what you were using to predict the weather
- What strategies were you using?

Specific Qs (using pictures of fractals)

*What percentage of the time were the following associated with a SUNNY outcome? Answer from 0-100. If unsure, please make your best guess!

Fractal 1 on the left (***regardles**s of the identity of the* fractal *in the centre*)

Fractal 1 on the right (***regardless** of the identity of the* fractal *in the centre)*

Fractal 2 on the left (***regardless** of the identity of the* fractal *in the centre)*

Fractal 2 on the right (***regardless** of the identity of the* fractal *in the centre)*

*What percentage of the time were the following associated with a SUNNY outcome? Answer from 0-100. If unsure, please make your best guess!

Fractal 1 AND fractal 3 (***regardles**s of position)*

Fractal 1 AND fractal 4 (***regardless** of position)*

Fractal 2 AND fractal 3 (***regardless** of position)*

Fractal 2 AND fractal 4 (***regardless** of position)*

Supplemental References

Cohen, N. J., and Eichenbaum, H. (1993). Memory, Amnesia and the Hippocampal System (Cambridge, MA, MIT Press).

Kumaran, D., Hassabis, D., Spiers, H. J., Vann, S. D., Vargha-Khadem, F., and Maguire, E. A. (2007). Impaired spatial and non-spatial configural learning in patients with hippocampal pathology. Neuropsychologia *45*, 2699-2711.

Mayes, A., Montaldi, D., and Migo, E. (2007). Associative memory and the medial temporal lobes. Trends Cogn Sci *11*, 126-135.

McClelland, J. L., McNaughton, B. L., and O'Reilly, R. C. (1995). Why there are complementary learning systems in the hippocampus and neocortex: insights from the successes and failures of connectionist models of learning and memory. Psychol Rev *102*, 419-457.

Rescorla, R. A., and Wagner, A. R. (1972). A theory of pavlovian conditioning: variations in the effectiveness of reinforcement and nonreinforcement. In Classical Conditioning II, A. H. Black, and W. F. Prokasy, eds. (New York, USA, Appleton-Century-Crofts), pp. 64-99.

Smith, A. C., Frank, L. M., Wirth, S., Yanike, M., Hu, D., Kubota, Y., Graybiel, A. M., Suzuki, W. A., and Brown, E. N. (2004). Dynamic analysis of learning in behavioral experiments. J Neurosci *24*, 447-461.

Watkins, C., and Dayan, P. (1992). Q-Learning. Machine Learning *8*, 279-291.

Wirth, S., Yanike, M., Frank, L. M., Smith, A. C., Brown, E. N., and Suzuki, W. A. (2003). Single neurons in the monkey hippocampus and learning of new associations. Science *300*, 1578-1581.
